# Conformational effects of *N*-glycan core fucosylation of immunoglobulin G Fc region on its interaction with Fcγ receptor IIIa

**DOI:** 10.1038/s41598-017-13845-8

**Published:** 2017-10-23

**Authors:** Yoshitake Sakae, Tadashi Satoh, Hirokazu Yagi, Saeko Yanaka, Takumi Yamaguchi, Yuya Isoda, Shigeru Iida, Yuko Okamoto, Koichi Kato

**Affiliations:** 1 Graduate School of Science, Nagoya University, Nagoya, Aichi 464-8602 Japan; 2 Graduate School of Pharmaceutical Sciences, Nagoya City University, 3-1 Tanabe-dori, Mizuho-ku, Nagoya, Aichi 467-8603 Japan; 3 Institute for Molecular Science and Okazaki Institute for Integrative Biosciences, National Institutes of Natural Sciences, 5-1 Higashiyama, Myodaiji, Okazaki, Aichi 444-8787 Japan; 4 School of Materials Science, Japan Advanced Institute of Science and Technology, 1-1 Asahidai, Nomi, Ishikawa, 923-1292 Japan; 5 Research Functions Unit, R&D Division, Kyowa Hakko Kirin Co., Ltd, 3-6-6 Asahi-machi, Machida-shi, Tokyo, 194-8533 Japan; 6 Information Technology Center, Nagoya University, Nagoya, Aichi 464-8601 Japan; 7 Structural Biology Research Center, Graduate School of Science, Nagoya University, Nagoya, Aichi 464-8602 Japan; 8 Center for Computational Science, Graduate School of Engineering, Nagoya University, Nagoya, Aichi 464-8603 Japan; 9 JST-CREST, Nagoya, Aichi 464-8602 Japan

## Abstract

Antibody-dependent cellular cytotoxicity (ADCC) is promoted through interaction between the Fc region of immunoglobulin G1 (IgG1) and Fcγ receptor IIIa (FcγRIIIa), depending on *N*-glycosylation of these glycoproteins. In particular, core fucosylation of IgG1-Fc *N*-glycans negatively affects this interaction and thereby compromises ADCC activity. To address the mechanisms of this effect, we performed replica-exchange molecular dynamics simulations based on crystallographic analysis of a soluble form of FcγRIIIa (sFcγRIIIa) in complex with IgG1-Fc. Our simulation highlights increased conformational fluctuation of the *N*-glycan at Asn162 of sFcγRIIIa upon fucosylation of IgG1-Fc, consistent with crystallographic data giving no interpretable electron density for this *N*-glycan, except for the innermost part. The fucose residue disrupts optimum intermolecular carbohydrate-carbohydrate interactions, rendering this sFcγRIIIa glycan distal from the Fc glycan. Moreover, our simulation demonstrates that core fucosylation of IgG1-Fc affects conformational dynamics and rearrangements of surrounding amino acid residues, typified by Tyr296 of IgG1-Fc, which was more extensively involved in the interaction with sFcγRIIIa without Fc core fucosylation. Our findings offer a structural foundation for designing and developing therapeutic antibodies with improved ADCC activity.

## Introduction

Rapid progress in antibody engineering has enabled a variety of biopharmaceutical applications of antibodies for therapeutic treatment of autoimmune diseases, cancer, and septicemia. Indeed, over 50 recombinant monoclonal antibodies have been approved in the US and EU as drugs against various cancers and chronic diseases. Furthermore, antibody-based therapeutics currently account for most recombinant proteins in clinical use, over 300 monoclonal antibody candidates have entered clinical trials, and approximately 70 monoclonal antibody products are expected to be on the market by 2020^1^. Among these applications, antitumor treatment with therapeutic antibodies is primarily based on antibody-dependent cellular cytotoxicity (ADCC), which is promoted through interaction between the Fc region of immunoglobulin G1 (IgG1) and Fcγ receptor IIIa (FcγRIIIa) on the surface of immune effector cells, such as natural killers and macrophages^2^.

The Fc region of IgG1 possesses one conserved *N*-glycosylation site (Asn297) in each C_H_2 domain, where a bi-antennary complex-type oligosaccharide is expressed with microheterogenities resulting from the presence or absence of non-reducing terminal fucose, galactose, and sialic acid residues. These *N*-glycans contribute to structural integrity of the FcγR-binding sites located in the hinge-proximal region of the C_H_2 domains and are therefore essential to expression of the FcγR-mediated effector functions of IgG1, including ADCC^3–5^. Interestingly, core fucosylation of Fc *N*-glycans has a critical negative effect on the interaction of IgG1 with FcγRIIIa and its consequent ADCC^4,6–12^. Namely, removal of the core fucose residue from Fc glycans causes dramatic enhancement of ADCC through improved IgG1-FcγRIIIa interaction.

Crystallographic studies have indicated that binding of IgG1-Fc to FcγRIIIa is mediated not only by protein-protein interactions but also by carbohydrate-protein and carbohydrate-carbohydrate interactions^13–15^. In this binding mode, one of two Fc glycans extensively contacts an *N*-glycan at Asn162 of FcγRIIIa, suggesting that core fucosylation causes steric hindrance against intermolecular carbohydrate-carbohydrate interactions and thereby negatively affects FcγRIIIa binding.

In this paper, by using molecular dynamics (MD) simulations based on crystal structures, we characterize the interaction between IgG1-Fc and the extracellular region of FcγRIIIa in solution, especially focusing on conformational dynamics of their *N*-glycans. To efficiently obtain sampling data regarding nonfucosylated and fucosylated IgG1-Fc complexed with FcγRIIIa, we employed replica-exchange molecular dynamics (REMD) method^16^, which has been demonstrated to be useful for exploration of conformational spaces of oligosaccharides^17–20^. The effects of core fucosylation of IgG1-Fc glycans on the intermolecular carbohydrate-carbohydrate interaction are discussed, based on the simulation results.

## Results and Discussion

### Crystal structures of fucosylated IgG1-Fc complexed with FcγRIIIa

**Figure 1:**
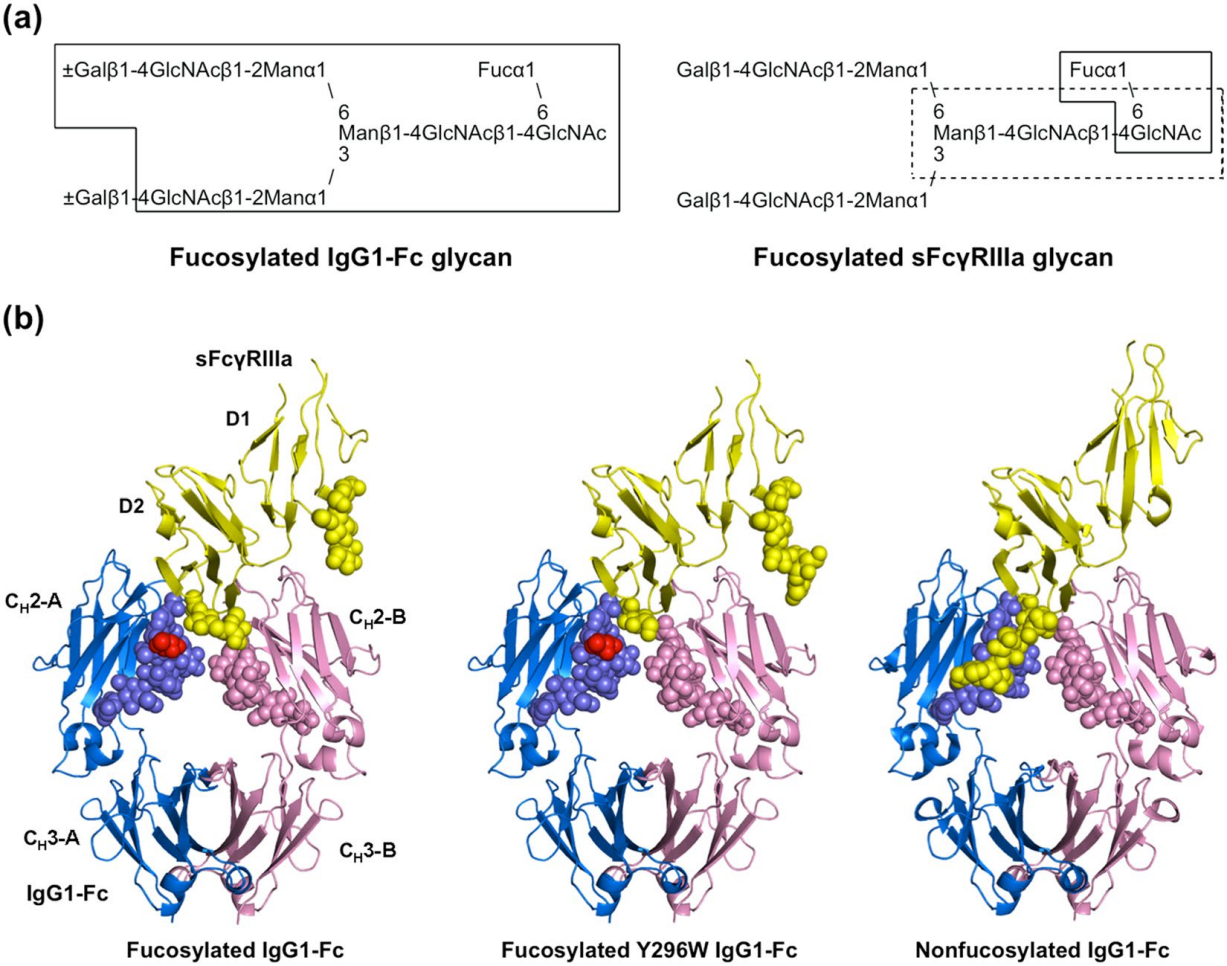
Crystal structures of IgG1-Fc/sFcγRIIIa complexes. (**a**) Schematic representation of *N*-glycans expressed on fucosylated wild-type IgG-Fc (left) and sFcγRIIIa (right) used for crystallization, showing the nomenclature of oligosaccharide residues and branches. Sugar residues that gave interpretable electron densities are surrounded by solid polygons in Fc-Asn-297 *N*-glycans and sFcγRIIIa-Asn-162 *N*-glycans, whereas those in the sFcγRIIIa-Asn-45 *N*-glycan are indicated by a dashed box. (**b**) Crystal structures of Fc fragments in complex with the bis-*N*-glycosylated soluble form of sFcγRIIIa: left, fucosylated wild-type IgG1-Fc; center, fucosylated Y296W IgG1-Fc; right, nonfucosylated wild-type IgG1-Fc (PDB code: 3AY4). Chain A of Fc fragment, chain B of Fc fragment, and sFcγRIIIa are colored by marine, pink, and yellow, respectively. Fucose residues are colored in red. Carbohydrate residues are represented as spheres.

To provide a structural basis for the negative effects of core fucosylation of IgG1-Fc glycans, we determined the crystal structure of fucosylated IgG1-Fc complexed with FcγRIIIa at 2.40-Å resolution. For crystallization, we used a soluble form of FcγRIIIa (sFcγRIIIa), which consisted of the extracellular domains and possessed two *N*-linked oligosaccharides at Asn45 and Asn162, while the remaining three *N*-glycosylation sites were mutationally eliminated. As expected, the overall structure of the complex formed between fucosylated IgG1-Fc and sFcγRIIIa was essentially identical to that formed with nonfucosylated Fc glycoform^14^, with a root-mean-square deviation value of 0.30 Å for the 578 superimposed C^α^ atoms (Fig. 1a).

In the crystal structure, the Asn297 glycans of Fc molecules showed well-defined electron density corresponding to a nonasaccharide containing a terminal galactose residue at the α1,6 arm. By contrast, *N*-glycans of sFcγRIIIa gave electron densities only for the reducing-terminal residues, i.e., GlcNAc1, GlcNAc2, and Man3 at Asn45 and GlcNAc1 and Fuc at Asn162 (Fig. 1b). This is also in marked contrast to the observation of the same sFcγRIIIa in complex with the nonfucosylated Fc glycoform, in which the Asn162 glycan is extensively involved in interactions with the Fc glycan^13–15^. Because the sFcγRIIIa glycoprotein used in the crystallization had a homogeneous glycoform exclusively exhibiting the bi-antennary complex-type oligosaccharide Gal_2_GlcNAc_2_Man_3_GlcNAc_2_(Fuc), the ambiguous electron density of the Asn162 glycan of sFcγRIIIa indicates its conformational disorder, presumably because the intermolecular carbohydrate-carbohydrate interaction was weakened in the presence of the core fucose residue of the Fc glycan^4,6–12^. This is consistent with the previously reported crystal structure formed between the fucosylated IgG1-Fc glycoform and sFcγRIIIa exclusively expressing high-mannose-type oligosaccharides, which gave no interpretable electron densities except for the reducing terminal GlcNAc1 and GlcNAc2 at Ans45 and GlcNAc1, GlcNAc2, Man3, Man4, and Man4*′* at Asn162^13^. We also solved a 2.50-Å resolution crystal structure of the complex of sFcγRIIIa with a fucosylated IgG1-Fc with a Tyr-to-Trp mutation at position 296, which caused a two-fold increase in sFcγRIIIa-binding affinity, i.e., *K*_D_ = 1.3 × 10^−7^ (M)^15^. In this crystal structure, electron density of the Asn162 glycan was again largely unobservable. The missing electron densities of the sFcγRIIIa glycans in complex with fucosylated IgG1-Fc prompted us to characterize the conformational dynamics of the *N*-glycans of these two glycoproteins forming the complex in solution, using MD simulations.

### Exploration of *N*-glycan conformational spaces by MD simulations

In order to complement the missing conformational information of the sFcγRIIIa glycans, we performed MD simulations of the IgG1-Fc/sFcγRIIIa complex in solution. Moreover, to examine the structural impact of core fucosylation of IgG1-Fc, we compared the simulation results between fucosylated and nonfucosylated Fc glycoforms (Supplementary Videos S1 and S2). To adequately explore their conformational spaces, we performed REMD simulations. We used as the initial structure the crystal structure of nonfucosylated IgG1-Fc complexed with bis-glycosylated sFcγRIIIa. We also prepared the starting model of the fucosylated IgG1-Fc glycoform complexed with sFcγRIIIa to compare to the nonfucosylated one. Because the protein parts are essentially identical between the crystal structures in the fucosylated and non-fucosylated systems suggesting irrelevance of the overall protein motions, the protein backbone atoms were restrained by a harmonic potential during the calculation except for the amino acid residues in spatial proximity with each *N*-glycan, i.e., 233–239, 247–292, and 303–443 in Fc chain A, 230–239, 247–292, and 304–444 in Fc chain B, and 10–42, 48–53, 56–124, 130–160, and 165–174 in FcγRIIIa. The simulation time of the REMD simulation of 64 replicas was 30 ns for each replica (the total simulation time was thus 1.92 μs). The first half (15 ns per replica) of the simulation data were discarded for thermalization and the remaining half (15 ns per replica), which is referred to as the production run, were recorded for later analyses for both systems. This thermalization time of 15 ns is based on the time series of various distances as shown in Supplementary Fig. S1; the data appear to have reached thermal equilibrium around 15 ns.

**Figure 2:**
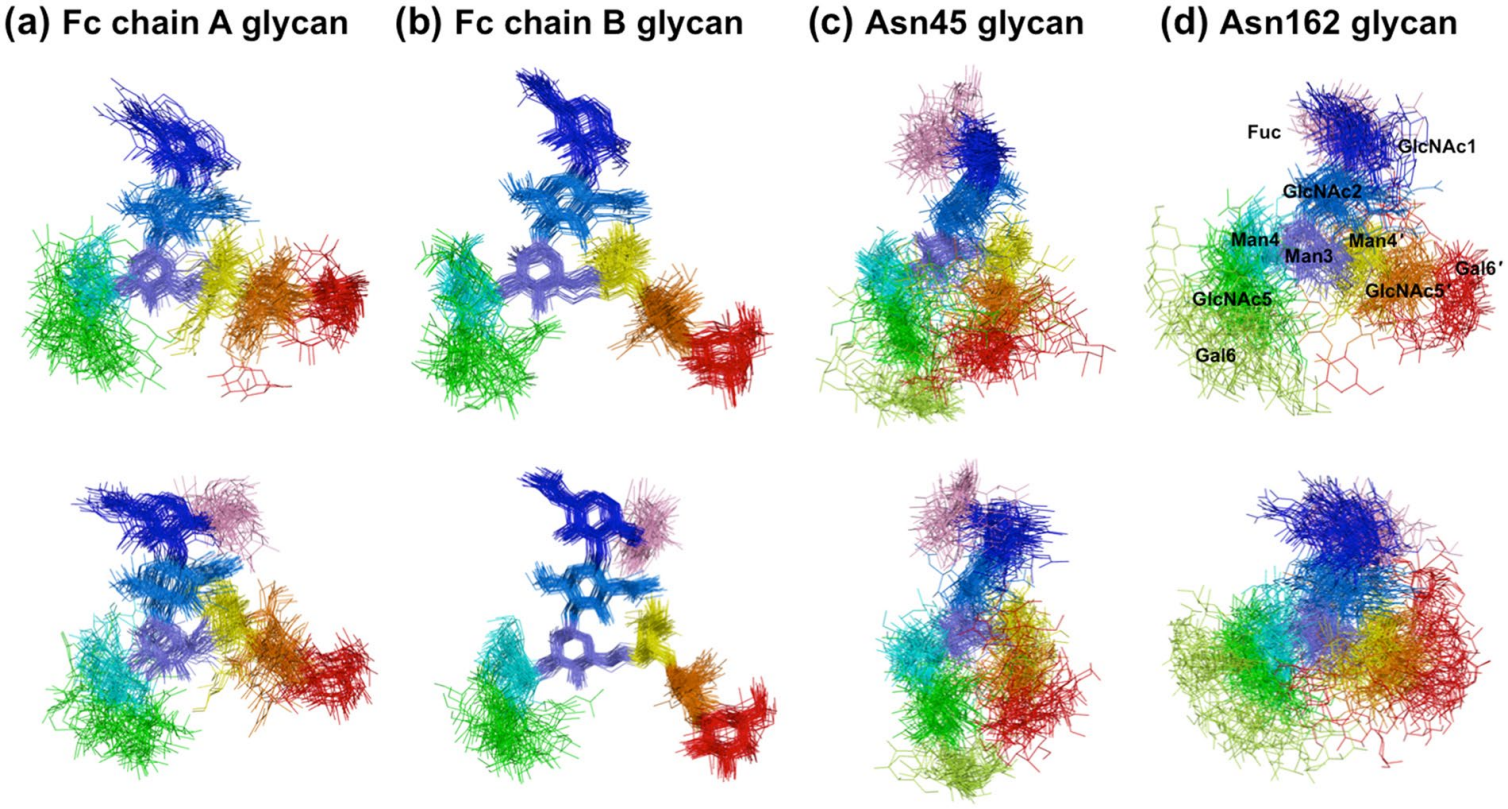
Superimposition of *N*-glycan conformers obtained from REMD simulation of IgG1-Fc/sFcγRIIIa complexes. One hundred conformers of the superimposed *N*-glycans of nonfucosylated and fucosylated systems are shown in the upper and lower panels, respectively: (**a**) Fc chain A glycan; (**b**) Fc chain B glycan; (**c**) Asn45 glycan; (**d**) Asn162 glycan. Each sugar residue is colored as follows: Fuc (pink); GlcNAc1 (blue); GlcNAc2 (marine blue); Man3 (slate); Man4 (cyan); Man4*′* (yellow); GlcNAc5 (green); GlcNAc5*′* (orange); Gal6 (lemon); Gal6*′* (red).

**Figure 3:**
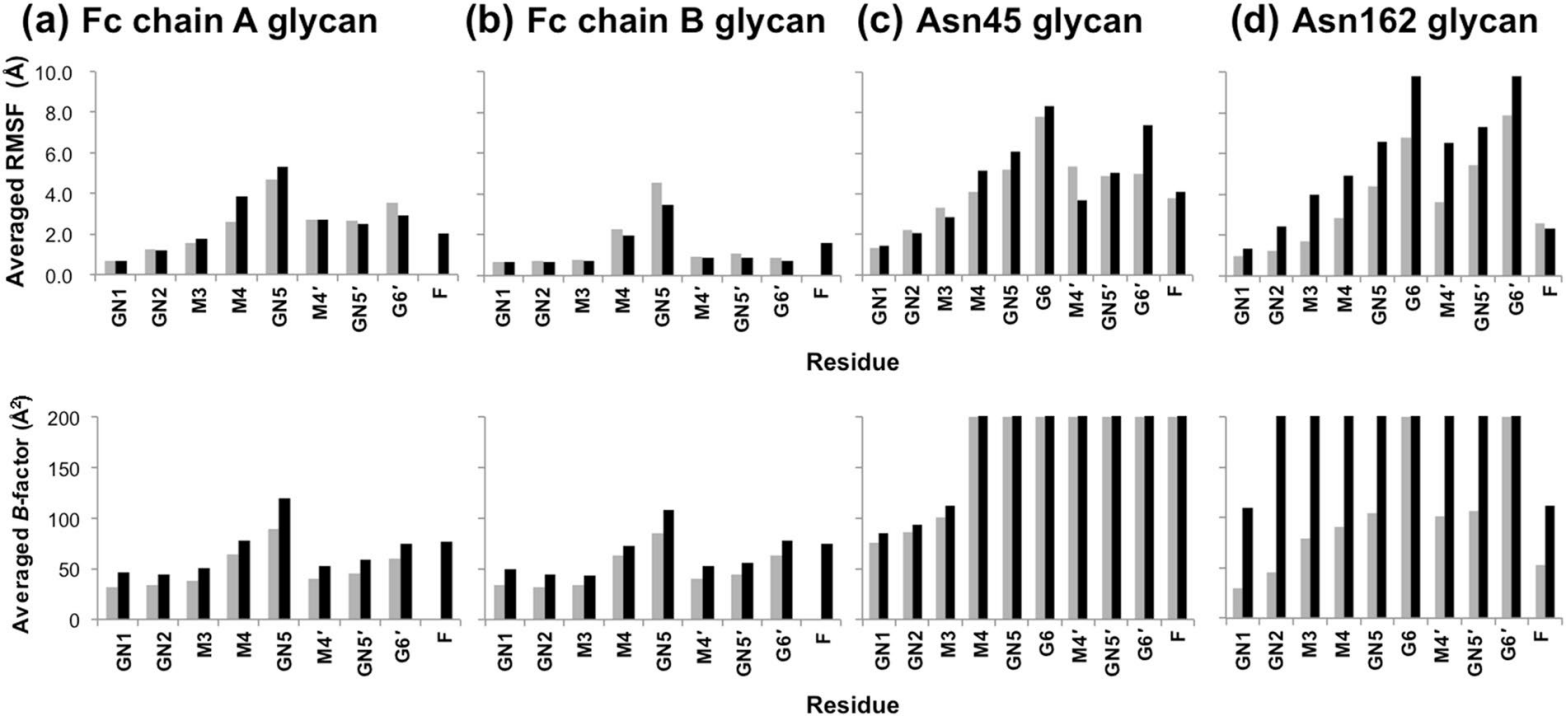
RMSF and crystallographic *B*-factor values for sugar residues of *N*-glycans of IgG1-Fc and FcγRIIIa. Averaged RMSF and crystallographic *B*-factor of each residue in the IgG1-Fc/FcγRIIIa complexes are shown in the upper and lower panels, respectively: (**a**) Fc chain A glycan; (**b**) Fc chain B glycan; (**c**) Asn45 glycan; (**d**) Asn162 glycan. The values of the nonfucosylated and fucosylated systems are shown as gray and black bars, respectively. The *B*-factor values of crystallographically disordered residues are indicated as 200 Å^2^. The residues are designated as follows: GlcNAc1 (GN1); GlcNAc2 (GN2); Man3 (M3); Man4 (M4); GlcNAc5 (GN5); Gal6 (G6); Man4*′* (M4*′*); GlcNAc5*′* (GN5*′*); Gal6*′* (G6*′*); Fuc (F).

For visualizing conformational fluctuations of the four *N*-glycans displayed on IgG1-Fc/sFcγRIIIa complexes with and without Fc fucosylation, superimposed snapshots derived from simulations performed at 300 K were compared. Among the four *N*-glycans, considerable differences, as exemplified by the widespread spatial distribution of the non-reducing terminal Gal6 residue, were found for the Asn162 glycan of sFcγRIIIa between the nonfucosylated and fucosylated systems (Fig. 2): the root mean square fluctuation (RMSF) value of the Asn162 glycan increased by about 1.8 Å, while the Asn45 glycan showed a higher RMSF, which were little affected by fucosylation of the IgG1-Fc glycans (Table 1). Both *N*-glycans of IgG1-Fc, which are packed within the two C_H_2 domains, were less mobile than the sFcγRIIIa glycans (Fig. 2a and b) and exhibited slightly decreased RMSF values with fucosylation (Table 1). Among the carbohydrate residues, outer residues, especially the Asn162 glycan in the fucosylated system, showed higher RMSF values as compared with inner residues (Fig. 3, upper panel). In addition, an increased tendency in the RMSF values is consistent with that of crystallographic *B*-factor or conformational disorder of the outer carbohydrate moiety (Fig. 3, lower panel). Thus, REMD-derived *N*-glycan dynamics and the crystallographic observation underscore increased fluctuation of the Asn162 glycan of sFcγRIIIa with fucosylation of IgG1-Fc. Therefore, we hereafter focused on the conformational dynamics of this sFcγRIIIa glycan.

**Table 1:** RMSF values for *N*-glycans of IgG1-Fc and FcγRIIIa.

*N*-glycan	Nonfucosylated (Å)	Fucosylated (Å)
Fc chain A glycan	2.5	2.6
Fc chain B glycan	1.5	1.3
Asn45 glycan	4.3	4.6
Asn162 glycan	3.7	5.5

### Effects of Fc fucosylation on intermolecular interactions

**Figure 4:**
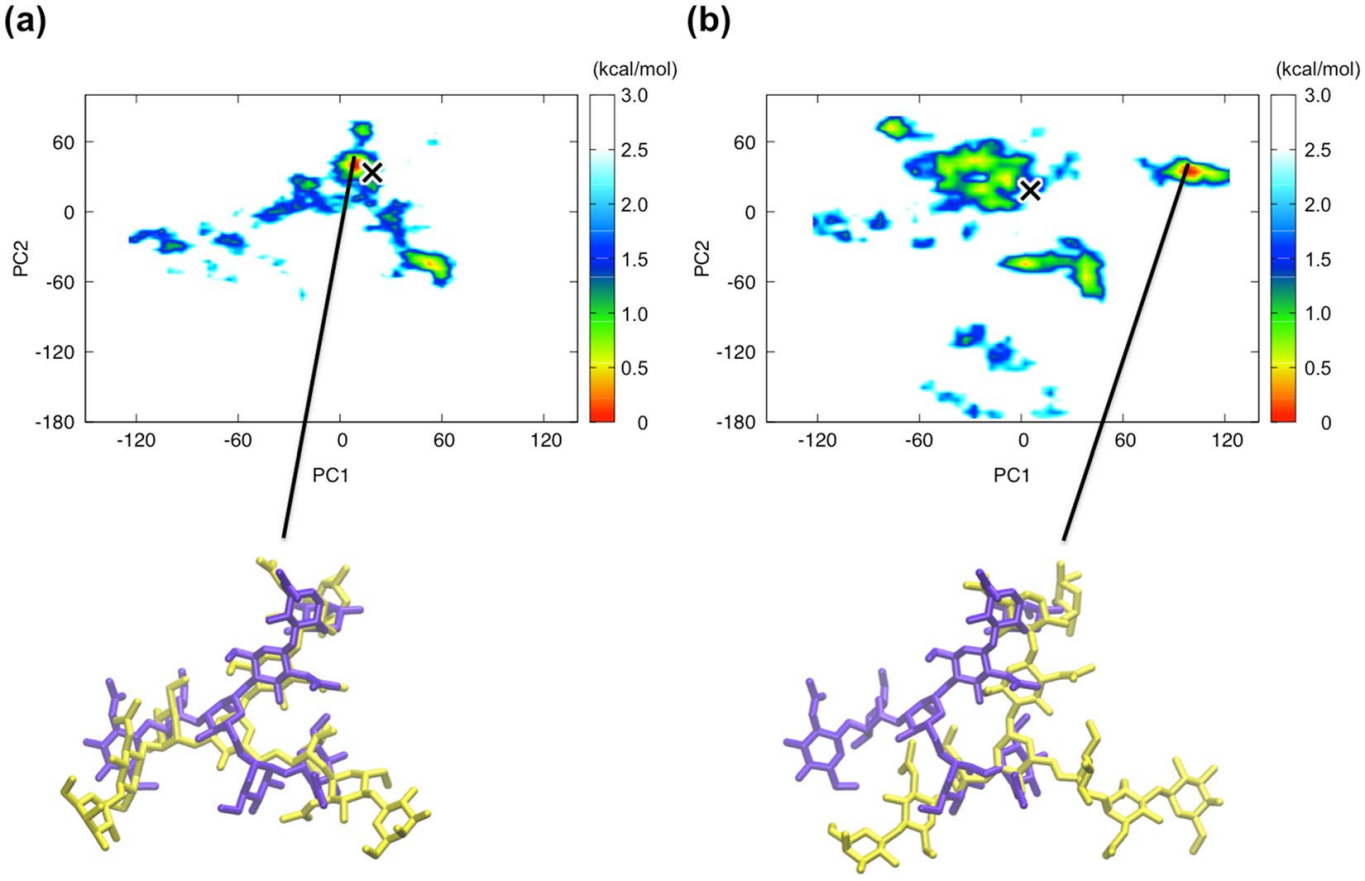
Principal component analysis of the simulation trajectory of the fucosylated and nonfucosylated systems. Free energy landscape along the first two principal components estimated from the trajectory of the nonfucosylated (**a**) and fucosylated (**b**) systems at 300 K. “X” represents position of the simulated structure which is most similar to the corresponding crystal structure. The Asn162 glycan crystal structure (purple) is superimposed onto the representative simulated structure (yellow) derived from the major conformational state in the simulation trajectory.

We calculated the free energy landscape along the first two principal components from the trajectory of the fucosylated and nonfucosylated systems (Fig. 4). The nonfucosylated system is characterized by one major conformational population, in which the Asn162 glycan showed a similar conformation to that seen in the crystal structure (marked as “X,” Fig. 4a). In contrast, the fucosylated system exhibited several distinctive conformational populations (Fig. 4b), among which the most populated conformational state of the Asn162 glycan was strikingly different from the crystal structure.

**Figure 5:**
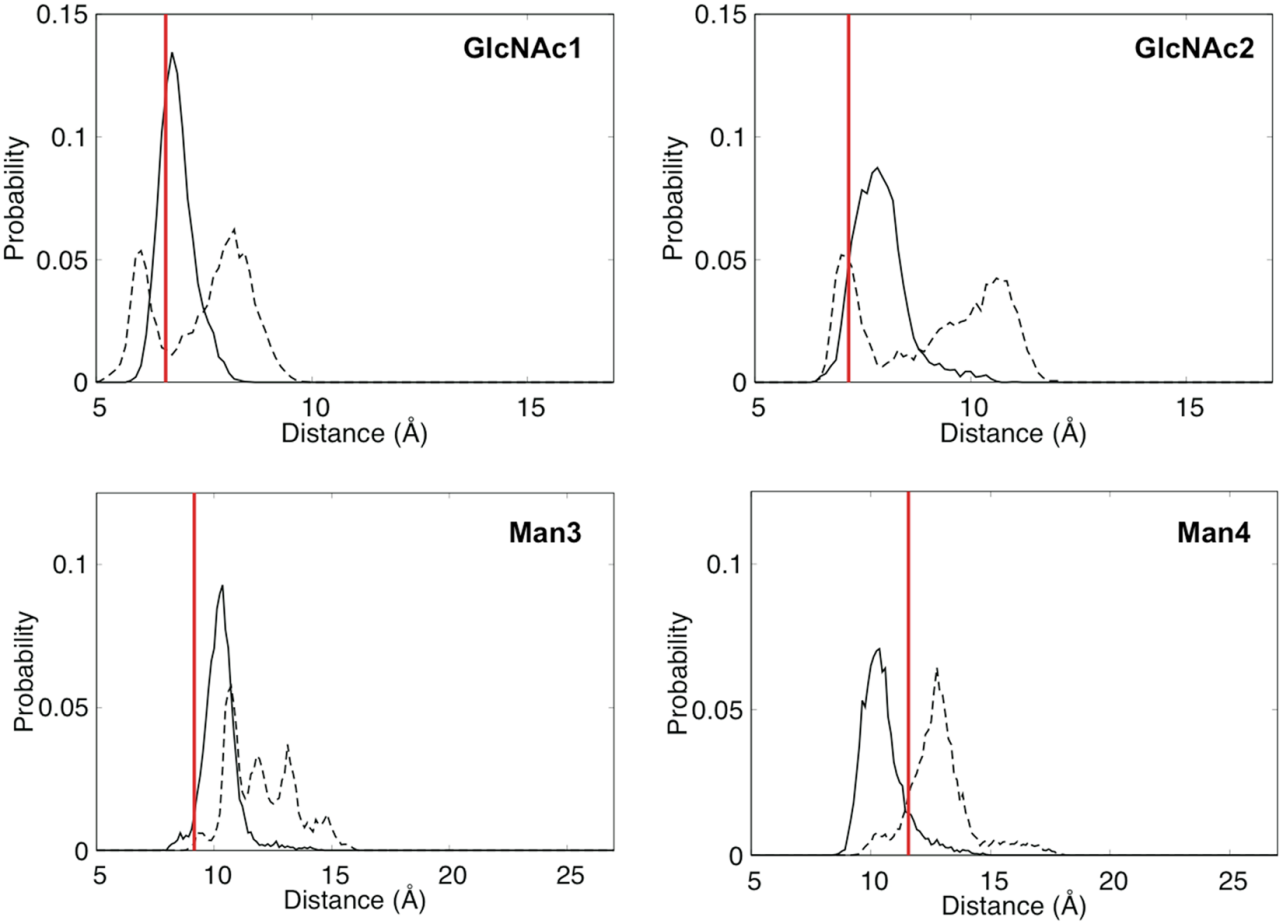
Distribution of distances between GlcNAc1 of IgG1-Fc and the inner residues (GlcNAc1, GlcNAc2, Man3, and Man4) of Asn162 glycan of sFcγRIIIa in nonfucosylated (solid line) and fucosylated (dotted line) systems. Red bars represent distances obtained by crystallographic analysis.

We compared distributions of distances between the centers of each residue of the sFcγRIIIa Asn162 glycan and that of GlcNAc1 of Fc chain A glycan, comparing the fucosylated and nonfucosylated forms (Fig. 5 and Supplementary Fig. S2). In the non-fucosylated system, this innermost residue of the Fc chain A glycan is in close proximity to the innermost part, i.e., GlcNAc1 and GlcNAc2, of the Asn162 glycan. The previous crystallographic data indicated that the Asn162 glycan of sFcγRIIIa is involved in intermolecular interactions with the IgG1-Fc glycan in its nonfucosylated form^13–15^. The crystal structure, in which the core and the Man α1-3 arm of the Asn162 glycan of sFcγRIIIa are in contact with the Fc glycan, corresponds in terms of these distances to the most highly populated state. By contrast, the REMD simulation indicates that these parts of the Asn162 glycan are more distal from the reducing terminus of the Fc chain A glycan in the fucosylated system. In particular, the reducing terminal GlcNAc1 and GlcNAc2 of the Asn162 glycan exhibit two populations in terms of the distance distribution (Fig. 5), in which the proximal population corresponds to the observed disaccharide in the crystal structure. Distance distribution of the neighboring residues including Man3 and Man4 is shifted to the distal positions in the fucosylated system (Fig. 5), whereas that of the outer residues, i.e., GlcNAc5, Gal6, Man4*′*, GlcNAc5*′*, and Gal6*′*, is little affected (Supplementary Fig. 2). The fucose attached to GlcNAc1 of Fc chain A occupied the space that was occupied by GlcNAc2 and Man3 residues of the sFcγRIIIa Asn162 glycan in the nonfucosylated form, which disrupts optimum intermolecular carbohydrate-carbohydrate interactions, rendering this sFcγRIIIa glycan dissociated from the Fc glycan.

**Figure 6:**
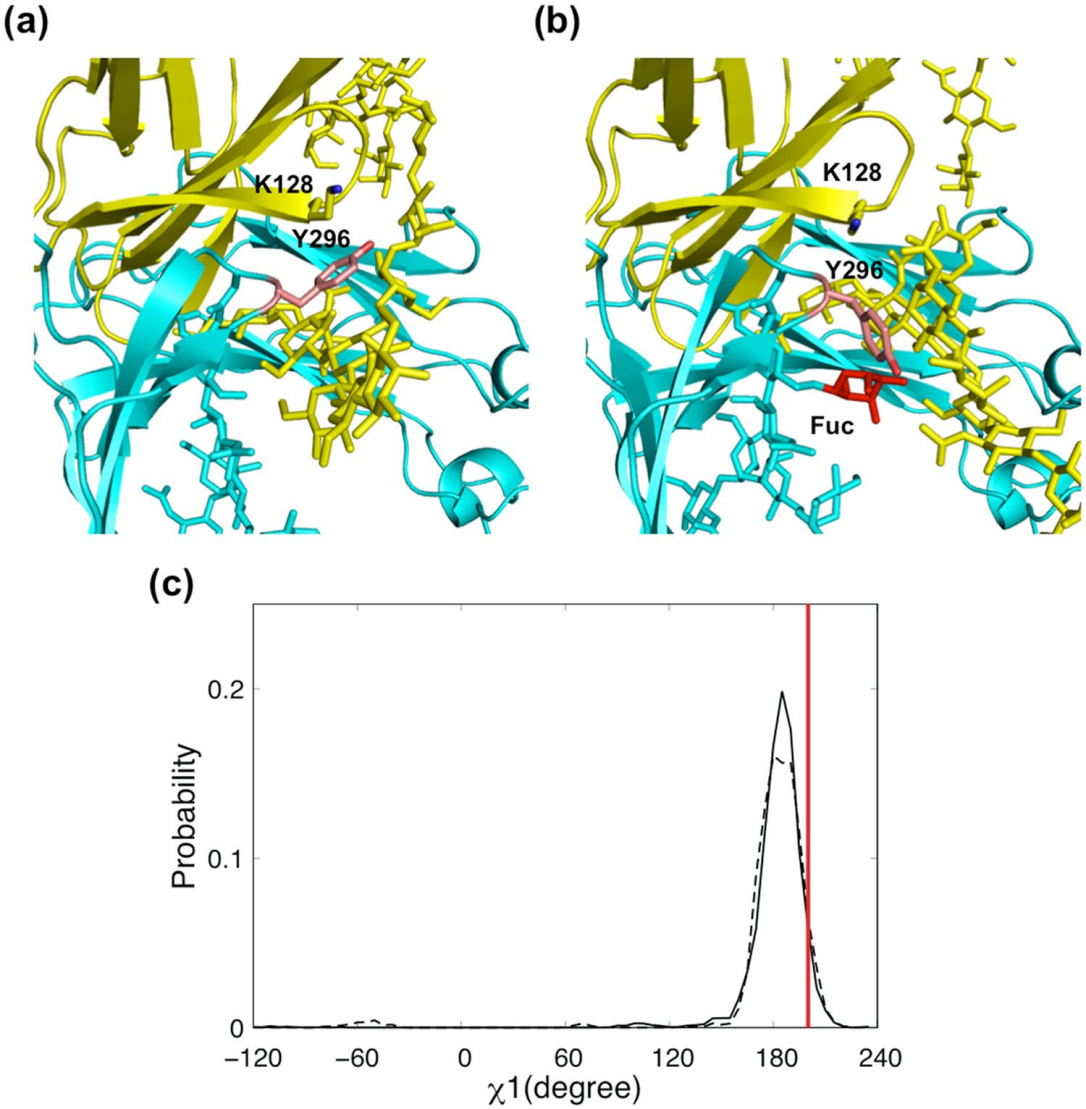
Conformational dynamics and rearrangements of Tyr296 of IgG1-Fc. Close-up views of the interaction interface between IgG1-Fc (cyan) and sFcγRIIIa (yellow) are shown: (**a**) nonfucosylated system; (**b**) fucosylated system. The representative simulated structures from the nonfucosylated and fucosylated systems, in which the χ1 dihedral angle of Tyr296 exhibited 186° and −55°, respectively, are shown as in Fig. 4(a) and Fig. 4(b), respectively. Sugar residues, Lys128 of sFcγRIIIa, and Asn297 and Tyr296 of IgG1-Fc are shown as sticks, while the fucose residue is colored red. (**c**) Distribution of χ1 dihedral angles of Tyr296 of IgG1-Fc in nonfucosylated (solid line) and fucosylated (dotted line) systems. Red bar represents the χ1 dihedral angle obtained by crystallographic analysis.

Our mutational data revealed the functional importance of Tyr296 of IgG1-Fc in its interactions with various Fcγ receptors, including FcγRIIIa^15^. In the crystal structure of the IgG1-Fc/sFcγRIIIa complex, the aromatic ring of Tyr296 is involved in interaction with the Asn162 glycan and Lys128 of sFcγRIIIa^13,14^. In this context, the results of our MD simulations indicate that core fucosylation of Fc glycan also intramolecularly perturbs the amino acid residues in close spatial proximity, as best exemplified by Tyr296, which neighbors the *N*-glycosylation site in Fc chain A. The aromatic ring of Tyr296 of nonfucosylated IgG1-Fc is sandwiched between the Asn162 glycan and the Lys128 side chain of sFcγRIIIa (Fig. 6a). In the presence of the IgG1-Fc core fucose, this major conformational state is significantly less populated, giving rise to divergent minor conformational states, which are exemplified by χ1 dihedral angle of −55° and also by direct contact with the fucose residue in Fc chain A glycan (Fig. 6b and c). Previous crystal structures have shown that, in fucosylated IgG1-Fc with or without sFcγRIIIa^21,22^, Tyr296 makes contact with the core fucose residue, while NMR data have indicated that this tyrosine residue undergoes conformational exchange in its absence^21^. The results of our REMD simulations thus indicate that the side-chain of Tyr296 becomes stabilized into a single conformational state that is accommodated in the sandwich arrangement upon defucosylation of IgG1-Fc glycans.

### Concluding remarks

Although oligosaccharides exhibit conformational dynamics in solution, their motional freedom can be partially restricted upon intra- and inter-molecular interactions^23,24^. The *N*-glycans of IgG1-Fc mostly gave unambiguous electron density in their crystal structures because they are packed between the two Fc subunits. By contrast, the *N*-glycans of sFcγRIIIa are displayed on its molecular surface and possess considerable degrees of motional freedom. In the crystal structures of the IgG1-Fc/ sFcγRIIIa complexes, clarity of electron density of the sFcγRIIIa Asn162 glycan depended on the presence or absence of the core fucose residue of the IgG1-Fc glycans. Our REMD simulations successfully extrapolated this missing information, revealing that the Asn162 glycan is stabilized in solution mainly through carbohydrate-carbohydrate interaction with the nonfucosylated form of Fc glycan, which, however, is sterically hindered by the core fucose of Fc. This conversion causes conformational rearrangements of the surrounding amino acid residues and the Asn162 glycan, which becomes more distal from Fc and rendered more mobile with Fc fucosylation.

Protein glycosylation is currently considered as one of the most critical factors in design and development of therapeutic antibodies. Our hybrid approach combines X-ray crystallography and REMD-based molecular simulations and provides a useful tool offering a structural foundation for creation of therapeutic antibodies with improved effector mechanisms.

## Materials and Methods

### Crystallization, X-ray data collection, and structure determination of fucosylated IgG1-Fc in complex with sFcγRIIIa

Binary complexes comprising fucosylated wild-type or Y296W IgG1-Fc fragment and bis-*N*-glycosylated sFcγRIIIa were purified as previously described^14^. The wild-type and Y296W IgG1-Fc/sFcγRIIIa complexes were concentrated to 10 mg/mL in 20 mM Tris-HCl (pH 7.5) and 100 mM NaCl and crystallized in a buffer containing 12% PEG20,000, 0.1 M MES (pH 6.5), and 4% Zwittergent 3–12 or 3–14 (Hampton Research), respectively, using a sitting drop vapor diffusion method at 20 °C. The crystals were cryoprotected with reservoir solution containing 15% glycerol and flash-cooled in liquid nitrogen. X-ray diffraction datasets were collected using synchrotron radiation (BL44XU, SPring-8, Japan) and processed using XDS^25^.

The 2.40 and 2.50 Å crystal structures of fucosylated wild-type and Y296W IgG1-Fc/sFcγRIIIa were solved by the molecular replacement method using MOLREP^26^, with the nonfucosylated wild-type form structure (PDB code: 3AY4) as a search model. Model fitting to the electron density map was conducted using COOT^27^. REFMAC5^28^ and PROCHECK^29^ were used for crystal structure refinement and stereochemical quality check, respectively. The crystal parameters and refinement statistics of the complex structures are summarized in Table 2. Molecular graphics were prepared using PyMOL software (https://www.pymol.org).

**Table 2:** Data collection and refinement statistics for the fucosylated IgG1-Fc/sFcγRIIIa complexes.

	Fucosylated IgG1-Fc/sFcγRIIIa	Fucosylated IgG1-Fc- Y296W/sFcγRIIIa
**Crystallographic data**		
Space group	*P*4_1_2_1_2	*P*4_1_2_1_2
Unit cell *a*/*b*/*c* (Å)	77.6/77.6/351.9	77.3/77.3/349.8
**Data processing statistics**		
Beam line	SPring-8 BL44XU	PF BL5A
Wavelength (Å)	0.90000	1.00000
Resolution (Å)	46.78–2.40 (2.54–2.40)	46.55–2.50 (2.65–2.50)
Total/unique reflections	487,882/43,217	512,558/37,895
Completeness (%)	99.5 (97.1)	99.7 (98.9)
*R*_merge_ (%)	10.4 (61.1)	7.7 (90.2)
*I*/σ (*I*)	14.2 (1.6)	23.7 (2.6)
*CC*_1/2_	0.998 (0.777)	0.999 (0.801)
**Refinement statistics**		
Resolution (Å)	20.0–2.40	20.0–2.50
*R*_work_/*R*_free_ (%)	23.3/28.1	22.0/27.0
Number of non-Hydrogen atoms		
Protein [Fc(A)/Fc(B)/FcR(C)]	1700/1712/1225	1714/1714/1225
Water	109	109
Sugar [Fc(A)/Fc(B)/FcR(C)]	110/110/63	110/110/78
R.m.s. deviations from ideal		
Bond lengths (Å)	0.010	0.011
Bond angles (°)	1.57	1.61
Ramachandran plot (%)		
Most favored regions	93.4	91.2
Additionally allowed regions	6.2	8.6
Generously allowed regions	0.4	0.2
Disallowed	0	0
Average *B*-factors (Å^2^)		
Protein [Fc(A)/Fc(B)/FcR(C)]	56.6/44.3/70.9	66.8/57.2/81.4
Water	47.3	54.5
Sugar [Fc(A)/Fc(B)/FcR(C)]	67.1/63.5/106.2	77.1/77.1/127.2

### Molecular dynamics simulations

For both systems, we employed REMD simulations to obtain efficiently sampled conformational data. The program package AMBER14^30^ was used with the force fields AMBER ff14SB^31^ and GLYCAM06^32^ for proteins and glycans, respectively, along with the TIP3P water model^33^. Total numbers of atoms of the nonfucosylated system and the fucosylated system were 96,797 and 96,323, respectively. As the initial structures for the simulations, we used the energy-minimized structures starting from the crystal structure of nonfucosylated IgG1-Fc complexed with bis-glycosylated sFcγRIIIa. For the missing residues of *N*-glycans, the energy-minimized conformations starting from the default conformations given by the GLYCAM06 were used. After the preparation of the initial conformations, we performed the equilibrium simulations for 4 ns with NPT Ensemble with periodic boundary conditions using the particle mesh Ewald method. Temperature was controlled with the Langevin thermostat with a collision frequency of 1 ps^−1^. Pressure regulation was achieved with isotropic position scaling with the Berendsen barostat with pressure around 1 atm and a pressure relaxation time of 1 ps. For all bonds involving hydrogens in the fragments, we used the SHAKE algorithm^34^ as constraint algorithm to carry out the simulations with 2.0 fs as the time step. The cut-off distance of 10 Å was used for non-bonded interactions. We also performed REMD simulations using a Langevin dynamics integrator with NVT Ensemble. The simulation times were 30 ns for each replica and each simulation used 64 replicas, in which 64 temperature values were distributed between 300 K and 400 K. Replica exchange of 32 pairs of temperatures was tried every 1000 MD steps. The simulations were first equilibrated for 15 ns followed by 15 ns production runs. Here we used as restraint a harmonic potential (*E*_restraint_ = *k*(***r*** − ***r***_0_)^2^, *k* = 10.0 kcal/mol, where ***r*** are atomic coordinates and ***r***_0_ are coordinates that were determined by experiments) for the backbone atoms in proteins, except the amino acids around the glycosylation sites and those in close spatial proximity with the *N*-glycans. Thus restrained amino acids residues were 233–239, 247–292, and 303–443 in Fc chain A, 230–239, 247–292, and 304–444 in Fc chain B, and 10–42, 48–53, 56–124, 130–160, and 165–174 in FcγRIIIa.

The root-mean-square fluctuation (RMSF) is defined for each atom by the following equation:

1 $${\rho }_{i}^{RMSF}=\sqrt{\langle {({{\boldsymbol{r}}}_{i}-\langle {{\boldsymbol{r}}}_{i}\rangle )}^{2}\rangle },$$

where ***r***_*i*_ is the coordinate of atom *i*, and <…> means the ensemble average. For Cartesian coordinate sets of *N* atoms fitted to the average coordinate in the principal component analysis, we calculated the variance-covariance matrix as

2 $${C}_{i,j}=\langle ({{\boldsymbol{r}}}_{i}-\langle {{\boldsymbol{r}}}_{i}\rangle )({{\boldsymbol{r}}}_{j}-\langle {{\boldsymbol{r}}}_{j}\rangle )\rangle ,$$

where ***r***_1_, …, ***r***_3*N*_ are mass-weighted Cartesian coordinates and 〈*r*_*i*_〉 is the average coordinate. Diagonalization of *C*_*i*,*j*_ results in 3 *N* eigenvectors *V*_*p*_ (*p* = 1, …, 3 *N*) and the corresponding eigenvalues λ_*p*_, which describe the modes of the collective motion and their respective amplitudes. The eigenvector is referred to as the *p*th principal component axis, and the principal components,

3 $${{\rm{\Phi }}}_{p}={{\bf{V}}}_{p}{\bf{R}},$$

are the projections of the data ${\bf{R}}={({{\boldsymbol{r}}}_{1}^{^{\prime} },\ldots ,{{\boldsymbol{r}}}_{3N}^{^{\prime} })}^{T}$, where ${{\boldsymbol{r}}}_{i}^{^{\prime} }={{\boldsymbol{r}}}_{i}-{{\boldsymbol{r}}}_{i}$, onto the eigenvectors *V*_*p*_. Using two principal components Φ_1_ and Φ_2_, the two-dimensional free energy landscape is obtained from

4 $$\Delta G({{\rm{\Phi }}}_{1},{{\rm{\Phi }}}_{2})=-{k}_{{\rm{B}}}T\,{\rm{l}}{\rm{n}}\,P({{\rm{\Phi }}}_{1},{{\rm{\Phi }}}_{2}),$$

where *P* is the probability density along the two principal components, *k*_B_ is Boltzmann’s constant, and *T* is the temperature.

### PDB accession codes

The coordinates and structural factors of the crystal structures of the fucosylated wild-type and Y296W IgG1-Fc/sFcγRIIIa complexes have been deposited in the Protein Data Bank under accession numbers 5XJE and 5XJF, respectively.

## Electronic supplementary material

Supplementary information

Supplementary Video S1

Supplementary Video S2
