# Supplementary material for: Conformational effects of N-glycan core fucosylation of immunoglobulin G Fc region on its interaction with Fcγ receptor IIIa
Source: Sci Rep. 2017 Oct 23;7:13780. doi: 10.1038/s41598-017-13845-8 (PMC5653758; doi:10.1038/s41598-017-13845-8)
Supplement: Supplementary file 1 — Supplementary information [file 41598_2017_13845_MOESM1_ESM.pdf]

Supplementary information

Conformational effects of *N*-glycan core fucosylation of immunoglobulin G Fc region on its interaction with Fcγ receptor IIIa

Yoshitake Sakae[1,*], Tadashi Satoh[2], Hirokazu Yagi[2], Saeko Yanaka[2,3], Takumi Yamaguchi[2,3,4], Yuya Isoda[5], Shigeru Iida[5], Yuko Okamoto[1,6,7,8,9], and Koichi Kato[2,3,*]

[1]Graduate School of Science, Nagoya University, Nagoya, Aichi 464-8602, Japan, [2]Graduate School of Pharmaceutical Sciences, Nagoya City University, 3-1 Tanabe-dori, Mizuho-ku, Nagoya, Aichi 467-8603, Japan, [3]Institute for Molecular Science and Okazaki Institute for Integrative Biosciences, National Institutes of Natural Sciences, 5-1 Higashiyama, Myodaiji, Okazaki, Aichi 444-8787, Japan, [4]School of Materials Science, Japan Advanced Institute of Science and Technology, 1-1 Asahidai, Nomi, Ishikawa 923-1292, Japan, [5]Research Functions Unit, R&D Division, Kyowa Hakko Kirin Co., Ltd, 3-6-6 Asahi-machi, Machida-shi, Tokyo 194-8533, Japan, [6]Information Technology Center, Nagoya University, Nagoya, Aichi 464-8601, Japan, [7]Structural Biology Research Center, Graduate School of Science, Nagoya University, Nagoya, Aichi 464-8602, Japan, [8]Center for Computational Science, Graduate School of Engineering, Nagoya University, Nagoya, Aichi 464-8603, Japan, [9]JST-CREST, Nagoya, Aichi 464-8602, Japan

[*]Correspondence to Koichi Kato (kkato@phar.nagoya-cu.ac.jp) and Yoshitake Sakae (sakae@tb.phys.nagoya-u.ac.jp)

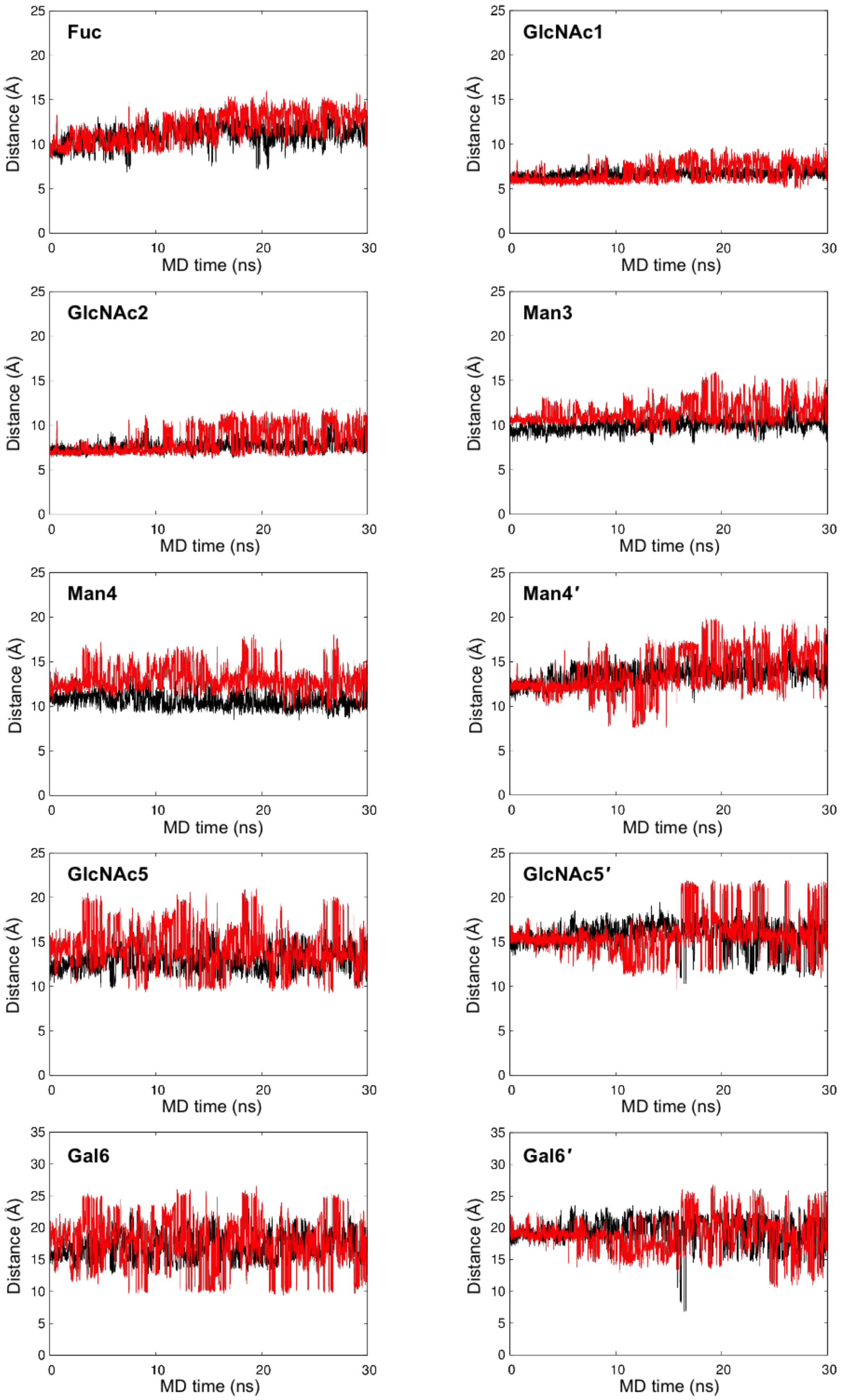

Supplementary Fig. S1.

Time series of the distances between GlcNAc1 of the Fc chain A glycan and the respective sugar residues (Fuc, GlcNAc1, GlcNAc2, Man3, Man4, Man4′, GlcNAc5, GlcNAc5′, Gal6, and Gal6′) of the Asn162 glycan of sFcγRIIIa in the nonfucosylated (black) and fucosylated (red) systems.

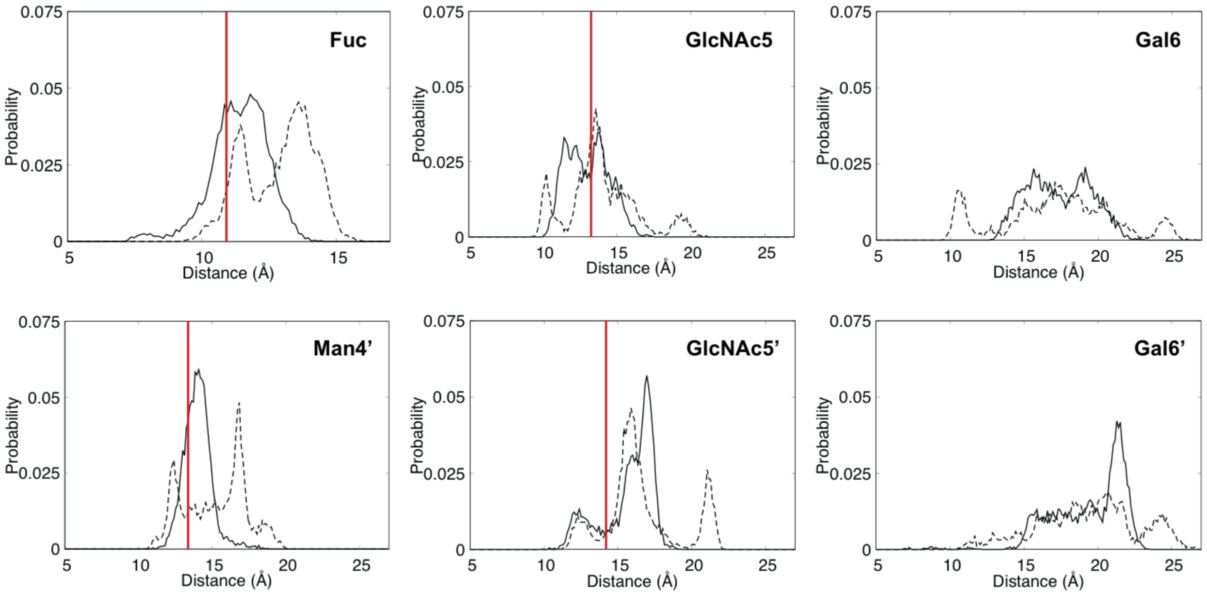

Supplementary Fig. S2. Distribution of distances between GlcNAc1 of IgG1-Fc and outer residues (Fuc, GlcNAc5, Gal6, Man4′, GlcNAc5′, and Gal6′) of the Asn162 glycan of sFcγRIIIa in nonfucosylated (solid line) and fucosylated (dotted line) systems. Red bars represent distances obtained by crystallographic analysis.

Supplementary Video S1. REMD simulation of the nonfucosylated IgG1-Fc/sFcγRIIIa complex. The *N*-glycans on chain A and B of Fc fragments and those on sFcγRIIIa are colored by marine, pink, and yellow, respectively. Carbohydrate residues are represented by sticks.

Supplementary Video S2. REMD simulation of the fucosylated IgG1-Fc/sFcγRIIIa complex. The *N*-glycans on chain A and B of Fc fragments and those on sFcγRIIIa are colored by marine, pink, and yellow, respectively. Carbohydrate residues are represented by sticks.
